# Imaging of Murine Whole Lung Fibrosis by Large Scale 3D Microscopy aided by Tissue Optical Clearing

**DOI:** 10.1038/s41598-018-31182-2

**Published:** 2018-09-06

**Authors:** Lorenzo F. Ochoa, Alexander Kholodnykh, Paula Villarreal, Bing Tian, Rahul Pal, Alexander N. Freiberg, Allan R. Brasier, Massoud Motamedi, Gracie Vargas

**Affiliations:** 1 Center for Biomedical Engineering, The University of Texas Medical Branch, Galveston, USA; 2 Department of Internal Medicine, The University of Texas Medical Branch, Galveston, USA; 3 Sealy Center for Molecular Medicine, The University of Texas Medical Branch, Galveston, USA; 4 Department of Pathology, The University of Texas Medical Branch, Galveston, USA; 5 Institute for Clinical and Translational Research, University of Wisconsin-Madison School of Medicine and Public Health, Madison, USA; 6 Department of Ophthalmology, The University of Texas Medical Branch, Galveston, USA; 7 Department of Neuroscience, Cell Biology and Anatomy, The University of Texas Medical Branch, Galveston, USA

## Abstract

Pulmonary fibrosis, characterized by excessive collagen deposition in the lungs, comprises a key and debilitating component of chronic lung diseases. Methods are lacking for the direct visualization of fibrillar collagen throughout the whole murine lung, a capability that would aid the understanding of lung fibrosis. We combined an optimized organ-level optical clearing (OC) approach with large-scale, label-free multiphoton microscopy (MPM) and second harmonic generation microscopy (SHGM) to reveal the complete network of fibrillar collagen in whole murine lungs. An innate inflammation-driven model based on repetitive poly(I:C) challenge was evaluated. Following OC, mosaic MPM/SHGM imaging with 3D reconstruction and whole organ quantitative analysis revealed significant differences in collagen deposition between PBS and poly(I:C) treated lungs. Airway specific analysis in whole lung acquisitions revealed significant sub-epithelial fibrosis evident throughout the proximal conductive and distal airways with higher collagen deposition in the poly(I:C) group vs PBS group. This study establishes a new, powerful approach based on OC and MPM/SHGM imaging for 3D analysis of lung fibrosis with macroscopic views of lung pathology based on microscopy and providing a new way to analyze the whole lung while avoiding regional sampling bias.

## Introduction

Chronic obstructive lung disease, a collective term that includes asthma, and chronic obstructive pulmonary disease (COPD), are highly prevalent conditions that produce significant morbidity. Approximately 26.5 million adults and children suffer from asthma^1^, and chronic lower respiratory disease, comprised mainly of COPD, is the third leading cause of death in the United States. Although etiologies of these diseases differ, they are commonly associated with inflammation-driven remodeling of structural elements of the lung. Pulmonary fibrosis is a common pathology of these lung diseases. Fibrosis results from an imbalance of synthesis and proteolytic degradation of the extracellular matrix. Depending on the environmental stimuli involved and structural cells affected, airway fibrosis can assume a variety of tissue distribution patterns^2^. In allergen-driven asthma, T lymphocytes and epithelial cells produce a characteristic pattern of sub-epithelial fibrosis in the lamina reticularis; an early and persistent finding^3^. Sub-epithelial fibrosis is produced by fibrillar collagen types I and III, downregulation of matrix metalloproteinases (MMPs), and proteoglycan deposition^4^. Moreover, the amount of lamina reticularis fibrosis correlates with increased obstruction and disease severity^5^. By contrast, intimal thickening and outer adventitial extracellular matrix deposition in COPD is associated with reduced expiratory airflow and exercise capacity. The molecular mechanisms, patterns of fibrosis, and relationships with other cellular events in remodeling are not completely understood. While a number of animal models have provided much of the current understanding of pulmonary fibrosis^6^, there is a lack of approach for direct study of fibrosis in the intact lung, particularly those that do not require tissue sectioning. Robust imaging approaches to identify patterns and characteristics of fibrosis throughout the full intact lung would greatly advance our understanding of this pathology, help assess disease risk factors, and contribute to identifying therapeutic targets.

Current approaches for preclinical study of lung fibrosis include whole-body imaging methods, histological approaches, and molecular biology methods. Whole-body imaging methods, including X-ray radiography, computed tomography (microCT), positron emission tomography (microPET), magnetic resonance imaging (MRI), and whole body bioluminescence/fluorescence by IVIS imaging provide *in vivo* indication of lung pathology at millimeter level resolution (50–100 µm for microCT) but do not provide direct assessment of the elements of fibrosis, namely collagen, and cannot delineate between lung inflammation and fibrosis^7–10^. Histology and immunohistology, used commonly in studies of fibrosis, provide subcellular visualization of microstructure including contrast of fibrillar collagen with staining. However, sampling is limited to 4–10 µm thick sections comprising small areas and is prone to regional sampling bias. This limited sampling of histology prohibits patterns of fibrosis to be characterized over large volumes and whole lung assessment is improbable as it would require sectioning across an entire organ^11^. Finally, molecular biology methods sample molecules directly associated with fibrosis, but are limited to fluids or digested tissue and thus lack spatial localization.

The nonlinear optical microscopy methods of multiphoton microscopy (MPM) and second harmonic generation microscopy (SHGM) provide imaging depths of hundreds of micrometers and in the lung have provided insights into the processes of inflammation and cancer both in *ex vivo* and intravital lungs^12–20^. MPM may be performed label-free with contrast for imaging three-dimensional lung structure based on intrinsic autofluorescence from cells and the extracellular matrix^13–15^. SHGM provides specific contrast to noncentrosymmetric molecules, of which fibrillar collagen is the primary source in lung^16^. Thus, with collagen overproduction and remodeling being central to fibrosis, SHGM provides a powerful option for fibrosis studies^17,18^. MPM and SHGM combine naturally and are often used together. With depths of imaging in the native lung to ~200 µm^16,19,20^ and the potential for image tiling or mosaicking (in which neighboring image stacks are acquired over large lateral areas) imaged volumes can far surpass volumes assessed by histology but are not sufficient for whole lung imaging due to depth limitations. However, recent developments in tissue optical clearing (OC) in which *ex vivo* tissues are rendered transparent through immersion in chemical agents, have made large scale microscopic imaging of full organs possible^21–24^.

OC includes a variety of chemical immersion approaches to reduce light scattering in tissue by index matching of tissue and interstitial components^25^. The method termed CLARITY which combines electrophoretic removal of lipids with immersion in clearing solvents was first introduced for full murine brain microscopy of neurons in fluorescent transgenic mice^26^. CLARITY was recently adapted and shown to render the lung transparent but was not applied for whole lung microscopy^27,28^. Lung CLARITY is a lengthy process, taking 13–17 days and leads to swelling with considerable expansion of organ volume^28^. A few studies have applied OC by direct solvent immersion with OC processing occurring in as little as a few days to a few hours, and imaged lung structures by confocal, MPM, or SHGM microscopy^21–23,29,30^. One limitation of solvent immersion methods is potential organ shrinkage >20–25%^25^. Three studies have demonstrated whole lung lobe microscopy, two employing confocal microscopy through intact lungs labeled with fluorophores and cleared with solutions of benzyl alcohol: benzyl benzoate or dibenzyl ether^23,30^, with Scott *et al*., first demonstrating imaging of the full lobe airway structure and visceral pleural nerves labeled with fluorescent antibodies^23^. Limitations in objective working distance limited imaging depths to 2 mm, thus lobes were separated and compressed between slides for imaging (details of depth were not discussed in Erturk *et al*.). In a recent study full lung fluorescence lightsheet microscopy with OC by immersion in tetrahydrofuran and dibenzyl ether (DBE) was demonstrated with the study focus on bronchus-associated lymphoid tissue^24^.

Thus far, whole lung microscopy of collagen for study of lung fibrosis has not been demonstrated. SHGM for imaging collagen has been performed on cleared lung, applied for regional lung imaging - with one study demonstrating 2 × 2 × 2 mm volume imaging of normal lung by MPM/SHGM^22^ while another achieved lateral lobe imaging but was limited by light attenuation to imaging depths of 397 µm^21^. Imaging depths needed to image uncompressed lung are 3–5 mm. Here, to address the gap in whole lung collagen imaging, which will enable studies of lung fibrosis, we have combined an optimized OC approach based on benzyl alcohol: benzyl benzoate (BABB) immersion that results in minimal change in tissue volume with whole-organ large scale microscopy by MPM/SHG mosaic imaging and image stitching. This combination of approaches (optimized OC sample processing, 3D MPM/SHGM mosaic imaging and stitching, and quantification) results in the ability to clear and image whole fixed lungs and reveal the global spatial distribution of fibrillar collagen throughout the organ at high resolution. The ability to identify and quantify fibrosis at the airway level and the volume of the lung is demonstrated.

## Methods

### Mouse model of lung fibrosis

A repetitive poly(I:C) challenge murine model of airway remodeling and subepithelial fibrosis served as the lung fibrosis model^31^. Animal experiments were performed according to the NIH Guide for Care and Use of Experimental Animals and approved by the University of Texas Medical Branch (UTMB) Institutional Animal Care and Use Committee (approval no. 1312058A). Male C57BL6/J mice (18 weeks old) purchased from The Jackson Laboratory (Bar Harbor, ME) were housed under pathogen-free conditions with food and water *ad libitum*. Mice were placed under light anesthesia [(ketamine (15 mg/kg bodyweight)/diazepam (1.5 mg/kg body weight)] for intranasal delivery of poly(I:C) to induce fibrosis. Intranasal poly(I:C) (500 μg/dose in 50 μL PBS) or an equivalent volume of PBS (50 μL) was administered every other day for a total of 15 doses (n = 4/group). Twelve days (12 d) after the last treatment at age 22 weeks, mice were anesthetized and lungs were harvested for OC and imaging or histology.

### Preparation of Lung extraction for OC

**Figure 1:**
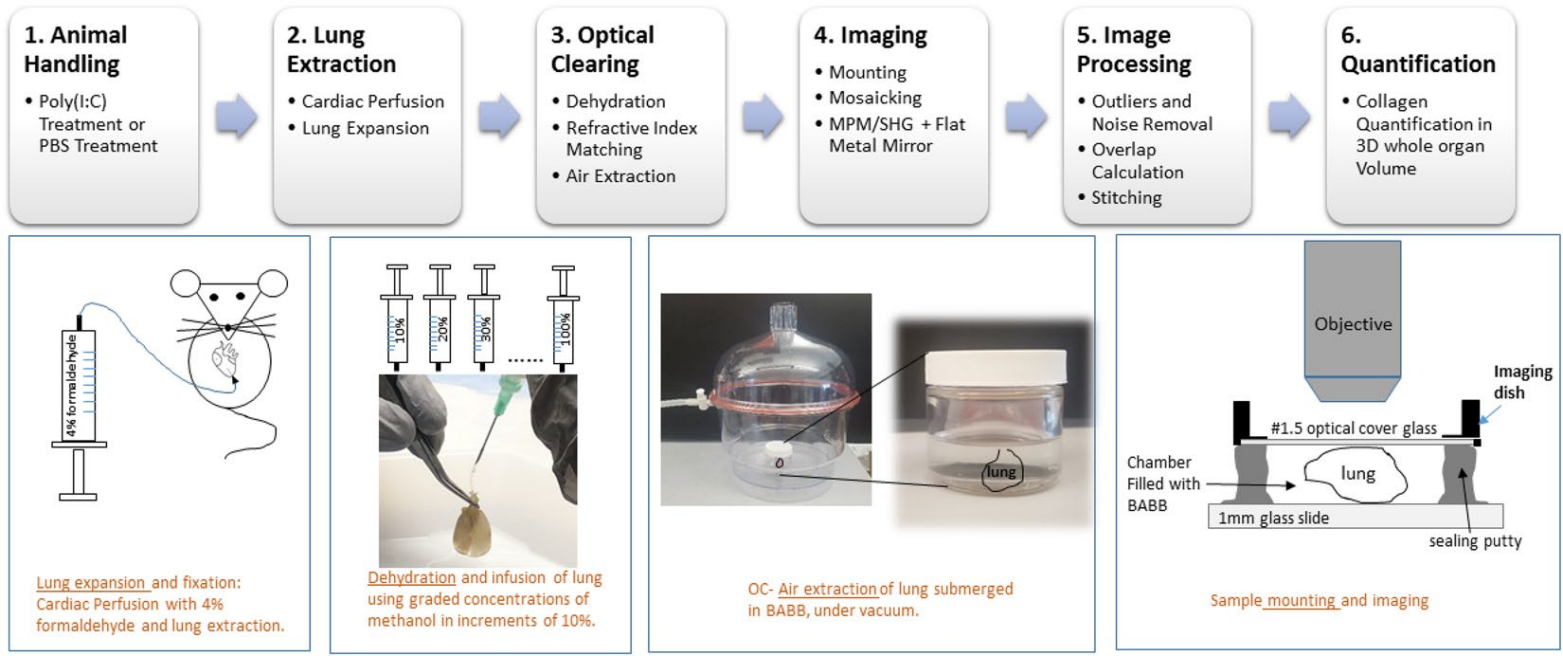
Whole Lung Optical Clearing and Imaging Workflow. 1. A murine poly(I:C) fibrosis model was used. Animals were treated with intranasal poly(I:C) to induce fibrosis or PBS as a control. 2. Lungs were extracted following cardiac perfusion in ~4 °C with PBS followed by 4% formaldehyde and were expanded post-extraction. 3. Lungs were treated with graded concentrations of methanol (second panel, bottom) for dehydration. Lungs were optically cleared by treatment with BABB for refractive index matching. Air microbubbles were removed by placing lungs in a vacuum chamber (third panel, bottom). 4. Samples were mounted (rightmost bottom panel) in an image chamber between a cover glass and a microscope slide. MPM/SHG mosaic imaging was performed by taking sequential z-stacks through the full depth of the lung (4 × 0.16 N.A. air objective, z interval: 5 µm n = 600–800 planes) across the entire organ with intrinsic tissue auto Fluorescence (MPM) and collagen SHG providing image contrast for the 3D volumetric acquisitions. 5–6. Image stack stitching was done by Fourier Transform Phase Correlation and quantification of collagen deposition (SHG) using 3D image analysis software.

Mice were anesthetized with 5% isoflurane using a nose cone for 3–5 minutes and reduced to 1.5% isofluroane. A transcardial perfusion was performed by opening the thoracic cavity exposing the heart and lungs. A blunt tip needle (Thermo Fisher Scientific, NC) was inserted in the right ventricle and a small incision was made in the left atrium (Fig. 1, lower leftmost panel). Transcardial perfusion was initiated with ~4 °C PBS (30–40 mL) followed by ~4 °C 4% formaldehyde (20–30 mL). While still attached *in situ*, lungs were then directly perfused via the trachea in order to avoid collapsing of the airways and maintain an expanded lung. Specifically, a blunt tip needle was inserted in the most superior position of the trachea, with the needle secured using a small bulldog clamp (World Precision Instruments, FL). Lungs were filled with 4% formaldehyde and the needle was removed leaving the bulldog clamp on the trachea. The lungs were removed and immersed in 4% formaldehyde (40 mL) overnight at 4 °C then subsequently placed in PBS (40 mL).

### Optical Clearing (OC)

An approach incorporating methanol for tissue dehydration followed by benzyl alcohol: benzyl benzoate (BABB) was employed for whole lung optical clearing, the protocol modified from previous lung OC studies^22,23^ to avoid significant shrinkage observed in such studies and to eliminate potential sources of scatter from air bubbles caught in the airway. For the dehydration step, graded concentrations of methanol starting with 10% methanol in PBS (v/v) in increments of 10% up to 100% methanol (room temperature) were infused into the lung via the cannulated trachea (Fig. 1, lower panels). Specifically, following overnight fixation in formaldehyde, described above, 10% methanol (5 ml) was slowly delivered via the trachea into the lung airways using a cannula tubing (10-gauge polyethylene cannula, Warner Instruments) attached to a 21-gauge needle on a syringe. The 5 ml solution was delivered manually over a period of approximately 1 minute. Next 20% methanol was delivered in the same manner (delay time of ~2 minutes), and so on in increments of 10% until 100% methanol was delivered. The 100% methanol infusion was repeated a second time and the lungs were placed in a conical tube containing fresh 100% methanol for 1 hour while placed on a shaker (Fisher Scientific CAT. NO. 22–363152). The purpose of this gradual increase in infusion solution was to avoid rapid volume changes in the lung during the methanol dehydration process. By slowly infusing the graded methanol concentrations through the trachea, large osmotic shifts were avoided. In pilot trials, we applied 50% methanol immersion as a first step as in two previous studies employing BABB clearing in lung^22,23^, but observed lung collapse and visible shrinkage thus we modified and optimized the dehydration step as described. Next, to render the lung transparent, it was infused with BABB (1:2 vol/vol mixture of benzyl alcohol [305197] and benzyl benzoate [B6630], Sigma-Aldrich) solution at room temperature as described above. Partial clearing of the lung became evident during this infusion with BABB delivered in approximately 1 minute. Following the 5 ml infusion with BABB, the lungs were immersed in 10 ml fresh BABB in a glass dish (QorPak, Bridgeville, PA) placed with a loose cap into a vacuum chamber overnight for removal of air bubbles seen in the transparent lungs. This step removed potential sources of light scatter from air bubbles trapped in the lung and allowed for complete BABB diffusion into tissue overnight. In preparation for imaging, samples were then mounted in a glass bottom dish (Willco Wells, UK) between a #1.5 coverglass on the imaging side and a 1 mm thick glass microscope slide on the opposing side (Fig. 1, lower rightmost panel), the two separated by moldable putty (Grainger, 5DLD8 or Bostik, 30840350) which served to seal the chamber and provided a spacing the thickness of the lung without compression. The chamber spacing was filled with fresh BABB immersion solution.

### Multiphoton and Second Harmonic Generation Microscopy (MPM/SHGM)

Multiphoton microscopy and second harmonic microscopy were performed on a Prairie Technologies Ultima IV laser scanning nonlinear optical microscope (Bruker, Middleton, WI) using a 4X 0.16 N.A. air objective (UPLSAPO 4X, Olympus) having a 2.2 µm lateral resolution and working distance of 13 mm and field of view (F.O.V.) of 2327 × 2327 µm as well as a 25 × 1.0 N.A. super-objective (XLSLPLN25XGMP, Olympus) with 0.4 µm lateral resolution and working distance of 8 mm and F.O.V. of 486 × 486 µm. The 4x objective was used for whole lung acquisitions while the 25X was used to collect regional areas. The sample holder was placed on the upright microscope stage below the objective with the coverglass facing the objective as shown in Fig. 1 (lower rightmost panel). Illumination for excitation of MPM autofluorescence and SHG was provided by a femtosecond laser (Mai Tai, Spectra Physics, Santa Clara, CA) tuned to 880 nm. Autofluorescence emission was collected using a 500–650 nm bandpass filter while SHG was collected at 440 nm using a notch filter with a bandwidth of 50 nm. To further increase the remitted nonlinear signal, a broadband metallic mirror (10D20ER.2, Newport Corp., Irvine CA) was placed under the sample chamber to reflect forward propagating light toward the objective, an approach shown to maximize SHG^32^.

To acquire whole lung image acquisitions (large scale microscopy), the 4X objective was used to acquire tiled image stacks through the full depth of the lung, acquiring neighboring image stacks in a tile pattern from left to right, row by row to form a mosaic tile pattern with the final number of tiles dependent on size of the lung. The increment between planes in depth for image stacks was 5 µm for the 4x acquisitions and total imaging depth was variable and ranged from 3–4 mm depending on the thickness of the lung. Acquisitions produced ~28,000–30,000 images each with each image stored as a 16-bit raw image (~10–15 GB of raw data per sample). Image stacks using the 25X objective were acquired with 2 µm step size and while image depths comprising full lung thickness were possible, regional imaging was generally limited to <1 mm due to time of acquisitions (typically 6 µs dwell time, 512 × 512).

### Image Processing and Analysis

Image processing and stitching of tiled acquisitions to create full lung mosaics were performed using FIJI/ImageJ^33^. Raw images acquired from the 12-bit sensor were stored as 16-bit tiffs (a standard storage protocol that incorporates zero padding of histogram values above 4095 (2^12-1^) when using a 12-bit sensor). Images were contrast stretched to remove zero padding prior to being converted to 8 bit images. A 2 × 2 median filter was applied to the image stacks. Stitching was performed with a 10% overlap of tiles having field of view of 2327.3 × 2327.3 µm, providing 232.73 µm of co-registration in both X and Y coordinates. The Fourier Transform phase correlation stitching method was applied using the ImageJ plugin *Grid/Collection Stitching*^34^. Quantification and 3D rendering were performed with Imaris software (Bitplane USA, Concord MA). After opening the mosaic stack, a 3D reconstruction was created and the *Surfaces* tool in Imaris was used to define the volume comprising SHGM. The *Surfaces* tool provides a threshold preview overlaid on the original image to allow for visual setting of a threshold value to match collagen structures visible in the SHG channel, then provides surface rendering. A threshold was determined visually for multiple stacks representing the dataset then kept the same between all lungs. The total SHG volume, representing the full lung fibrillar collagen content, was defined by surface rendering and calculated for the full lung using the volume analyzer in this tool. The total SHG volume was normalized to the total volume occupied by lung tissue, indicated from the volume of the MPM autofluorescence signal which was also processed with the “Surfaces” tool.

Volumetric analysis on individual airway types (primary bronchus, secondary bronchi, terminal bronchioles) was performed using ImageJ software (NIH). Specifically, for each airway type, non-branching segments of the airway conduit were identified visually and regions of interest chosen manually in the whole lung MPM/SHGM mosaic stack to isolate an image cube comprising the segment of airway conduit. Specifically, the *Rotated Rectangle* selection tool allowed for selection of laterally angled regions of interests for angled airways and the z dimension of the ROI cube was specified by choosing a plane immediately above the topmost surface and bottommost surface having SHG signal (the ImageJ *Slice Remover* tool was used to remove planes above and below stack of interest). The ROI cube lateral borders running parallel to the airway direction were also chosen immediately beyond termination of SHG signal. MPM (red) and SHGM (green) channels of the selected ROI image cube were separated and SHGM ROI stacks were manually thresholded (kept constant between samples) and binarized. The *Voxel Counter* plugin in ImageJ^33^ was used to calculate the volume of SHG-positive signal normalized to the ROI image cube. In this analysis, 6 airway segments were measured per each of the three airway types in each animal, and an average obtained per animal with a final average obtained per treatment group. As a complementary qualitative examination of collagen deposition in distal bronchioles, a visual count was made on the 3D lung view of the number of distal bronchioles surrounded by collagen in poly(I:C) vs control lungs seen using the *3D viewer* tool.

All processing was on a custom-built computer with Dual Quad-Core Xeon E5-2623 processors (10 MB Cache; 3.0 GHz; 128 GB of ECC RAM) with AMD Firepro W7100 graphics card.

### Histology

For histological examination, whole lungs were extracted without PBS perfusion. Extracted lungs were inflated under 25 cm H_2_O pressure with 10% (v/v) neutral buffered formalin through the tracheal cannula and immersed in 10% formalin solution (20X volume over the size of lungs) overnight. The next day, the whole lungs were replaced in fresh 10% formalin solution for another 48 hours and sent to UTMB Histopathology core for histological processing, where they were embedded into paraffin blocks, and cut into 5-μm sections. Slides were stained with Masson’s Trichrome to assess fibrotic changes. The histological images were taken on a standard microscope (Olympus IX71 using a Nikon Ds-Fi1 5-megapixel CCD (2560 × 1920 pixels).

### Statistics

Statistical analysis of total lung collagen volume between two groups (PBS vs. poly poly(I:C)) was performed using Student’s t-test (two-tailed) with p < 0.05 considered statistically significant. ANOVA Two Way Factor with Replication was performed for analysis of the three airway types (main stem bronchus, segmented bronchus, and terminal bronchioles) between PBS and poly(I:C) treated lungs. P values of <0.05 were considered statistically significant.

## Results

### Optimized Whole Lung OC with MPM/SHGM Mosaic Imaging

Figure 1 depicts the workflow of whole lung OC and large-scale microscopy. Optimized OC involved full lung inflation, a graded concentration dehydration step to avoid major size deviations, infusion of the lung with methanol and BABB to allow contact with the tissue from both the external and the internal airway surfaces, and the use of a vacuum chamber to remove air microbubbles which could contribute to light scattering in the lung. Tissue autofluorescence in MPM provided the contrast signal for microstructural assessment, while SHG provided contrast from fibrillar collagen in the extra-alveolar space.

**Figure 2:**
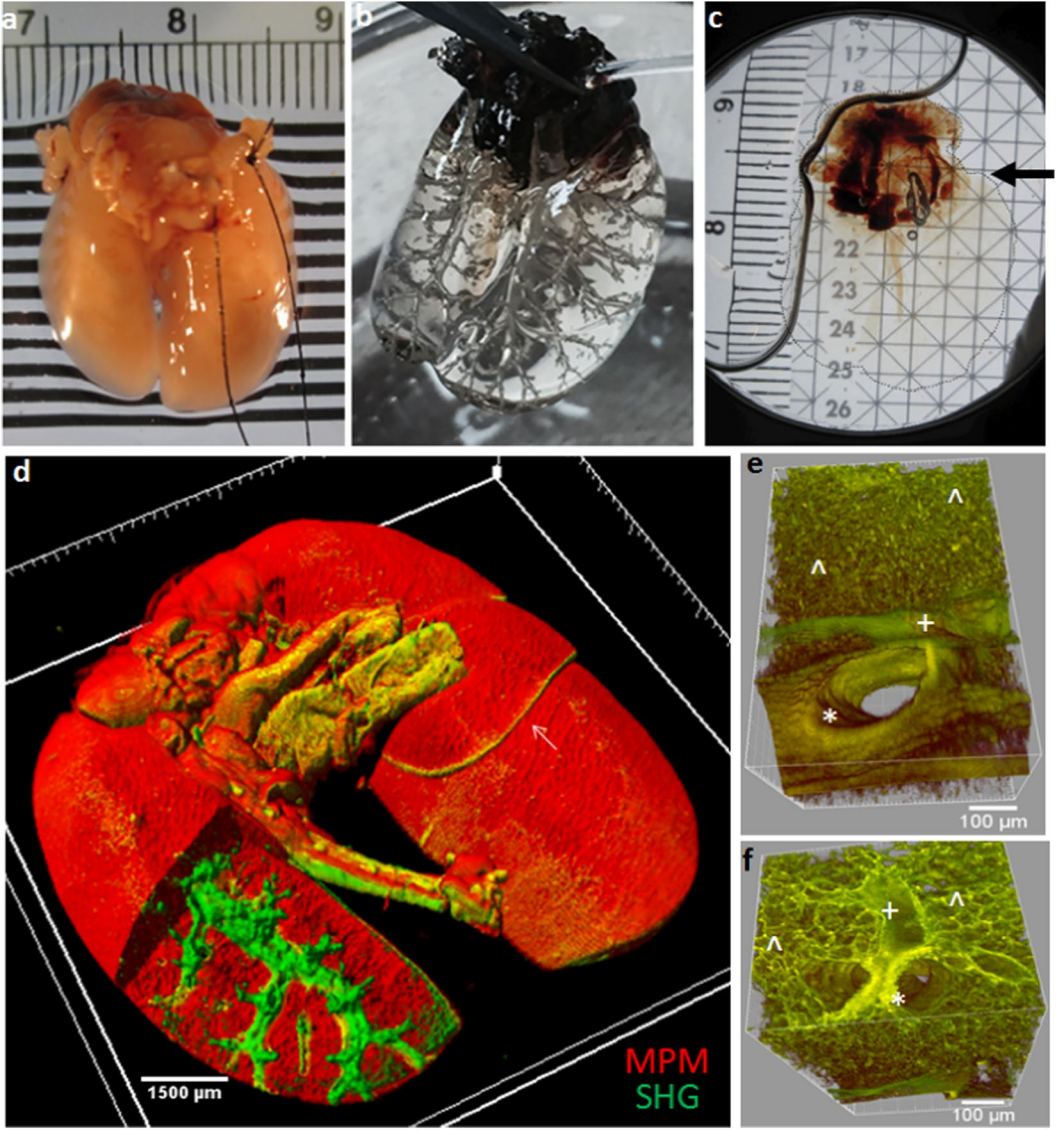
Optical Clearing and Large Scale Microscopy of Whole Lung. (**a**) Native murine lung following extraction (**b**) Intact lung following whole lung optical clearing. In this photograph, the lung is held in air following perfusion and immersion in BABB. To capture the contrast of the airway against the parenchyma, a cannula is used to perfuse the lung with fluid of slightly different refractive index to the BABB OC fluid. (**c**) Optically cleared lung infused with and immersed in BABB in a sample imaging chamber. A dotted line outlines the borders of the lung and the arrow points to area of cardiac structures that appear dark. (**d**) Volumetric (3D) representation of the entire murine lung obtained by mosaicking and stitching of MPM-SHGM image stacks using the 4 × 0.16 NA objective. Imaging was through the full depth of the lung with mosaicking (r x r arrangement) to acquire the entire organ, with MPM tissue autofluorescence shown in red and collagen SHG shown in green. The right lung shows multi- lobe structure (arrow). To create the cutout shown in (**d**), cropped areas of the red channel were removed from the left lung to reveal underlying sub epithelial collagen network surrounding airways. 3D reconstruction was by Imaris 3D analysis software. Reconstructed volume represents n = ~25,000 images. (**e**) Representative high resolution 25x MPM of lung regions showing a segment of a pulmonary bronchus (shown in false color). (**f**) MPM (25x) showing a segment of lung tissue with a vessel (+) near two converging bronchi (*) and surrounded by alveoli (^). (Epithelial cells (*), alveoli (^), and blood vasculature distribution (+)).

Representative images of a murine lung before and after OC are shown in Fig. 2. Freshly extracted native lung is highly turbid with typical color shown (Fig. 2a). Following infusion and immersion in BABB, the murine lung becomes fully transparent (Fig. 2b,c). In Fig. 2b, the transparent lung is held in air following perfusion and immersion in BABB. In this image, a cannula inserted into the trachea and used to infuse the lung with fluid of a slightly different refractive index to that of BABB allows the airways of the lung to appear with slight contrast relative to the lung parenchyma. However, once the lung is infused and fully immersed again in BABB (Fig. 2c) the airways are no longer distinguishable and the whole lung appears virtually invisible to the naked eye (a dotted line border delineates the edges of the lung against the solution in Fig. 2c). Here, only the cardiac structures that remain attached to the lung are evident, indicated by the arrow in Figs 2c. Fig. 2d shows the full depth three-dimensional MPM-SHGM reconstruction of the intact lung following mosaic imaging and tiling, with parenchymal autofluorescence in red and fibrillar collagen SHG in green. The right lung shows a multi-lobe structure as expected (arrow in Fig. 2d) with a single lobe in the left lung. A cutout of the left lung (removing a volume of the red channel) is made to reveal the intricate airway structure surrounded by fibrillar collagen. Select, high resolution (25x) regions of interest of multiphoton autofluorescence, shown in false color, illustrate airway morphology. A large airway (likely secondary bronchus) is shown lined with epithelial cells denoted by (*). Alveoli (^), and vasculature (+), is denoted by symbols (Fig. 2e,f). Lateral measurements in still images and photographs made on lungs that were dehydrated using the graded concentration application of methanol indicted +2.8 ± 1.7% change size, versus -16.9 ± 1.7% measured in lungs dehydrated with 100% methanol dehydration (5% shrinkage for 50% methanol). In the current study, lungs experienced a horizontal size change of approximately +6% (6.1% ± 1.5% for the lung shown in Fig. 1) after the full process of dehydration and clearing.

### Imaging Capabilities of MPM/SHGM in Optically Cleared Lungs

**Figure 3:**
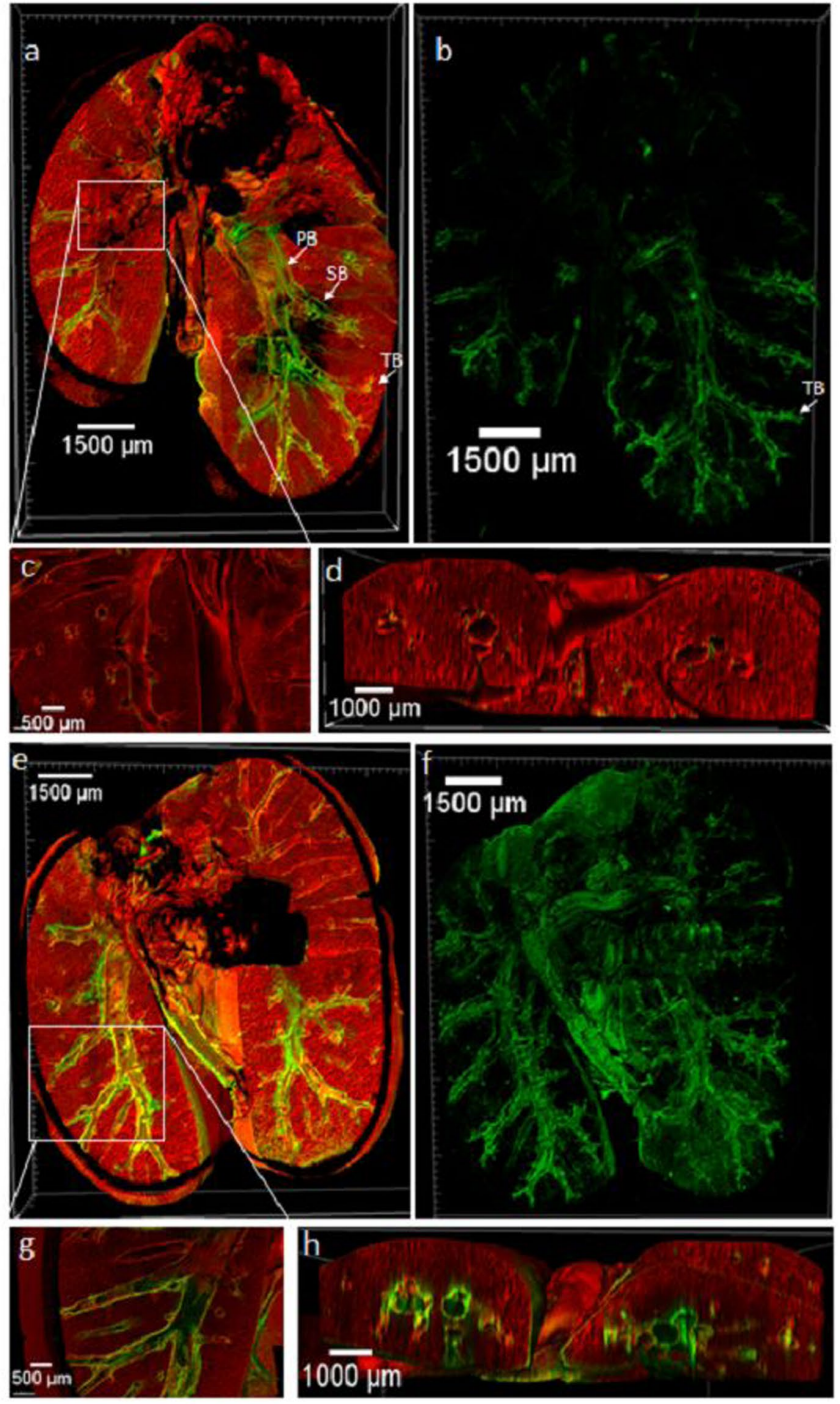
Large Scale MPM/SHG Microscopy Reveals Whole Lung Airway Structure and Airway Subepithelial Collagen Throughout the Full Lung. MPM/SHG representative 3D reconstructions of PBS and poly(I:C) treated lungs. PBS (**a**,**c**) and poly(I:C) (**e**,**g**) MPM/SHG 3D coronal cross-section of full lung. MPM autofluorescence is shown in red and fibrillar collagen SHG is shown in green. Representative 3D rendering of whole lung collagen network in PBS (**b**) and poly(I:C) (**f**) treated lungs. Close up of coronal (**c**) and transverse (**d**) cross-sections of PBS treated lungs. Close up of coronal (**g**) and transverse (**h**) cross-sections of poly(I:C) treated lung. Images show fibrotic collagen deposition surrounds airways in the subepithelial space (**g**,**h**). Primary bronchus (PB), Secondary bronchus (SB), Terminal bronchioles (TB) are designated in (**a**,**b**).

The capabilities of large scale MPM/SHGM mosaics with image stitching combined with optically cleared whole lungs are demonstrated in Fig. 3. Figure 3a–d show the ability to define the full lung structure including airways lined by subepithelial collagen, and the increase in collagen deposition found in the fibrosis model. 3D volumes of the SHG channel representing fibrillar collagen are shown in Fig. 3b,f for PBS control and poly(I:C) fibrosis model lungs. Transverse cut (Fig. 3d,h) renderings highlight and define the localization of fibrosis surrounding the airway to the subepithelial space.

### Comparison of MPM/SHGM with Histology

**Figure 4:**
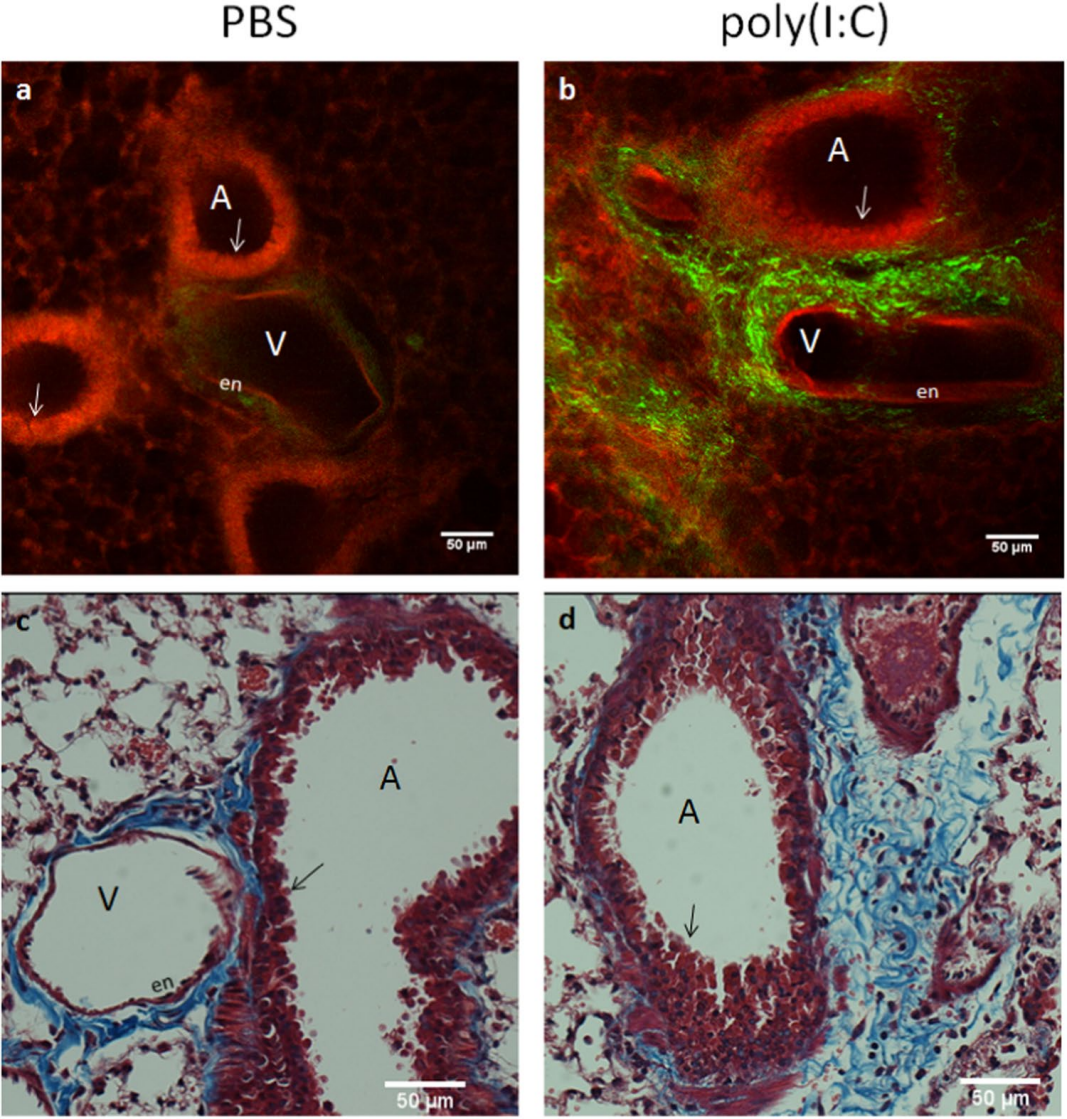
Collagen Deposition Revealed by MPM/SHG is Reflective of Deposition Seen in Histology. (**a**) MPM/ SHGM micrographs of PBS and (**b**) poly(I:C) treated lung showing airways and vessels surrounded by alveoli. (**c**) Histology with Masson’s Chrome staining for collagen shown at 20x for PBS and (**d**) poly(I:C). Enhanced collagen deposition is visible in the fibrosis model both in panels (**b**) MPM/SHG and (**d**) histology, with similarities in distribution and structure. Note the epithelial structure of airways in MPM micrographs (**b**) can be seen and similar to that of histology (arrows); Vessel endothelium(en).

The distribution of collagen near airways and vessels are further validated and appreciated in high resolution MPM/SHGM micrographs of Fig. 4 in PBS and poly(I:C) treated lungs. Airways (labeled, A) are defined by the presence of an epithelial lining visible due to MPM autofluorescence in red. Airway, vessel (V) and collagen morphology resembles that seen in histology in the lower panels shown at comparable scales (Fig. 4c,d). Vessels (labeled, V) are identified by the presence of thin endothelium. Airway subepithelial collagen is significantly increased in the poly(I:C) treated lung, as shown in MPM/SHGM micrographs (Fig. 4b vs. 4a), corroborated in histology (Fig. 4d vs. 4c) through Masson’s trichrome staining on tissue sections from comparable areas of PBS and poly(I:C) treated lungs.

### Whole Lung Fibrosis Assessment

**Figure 5:**
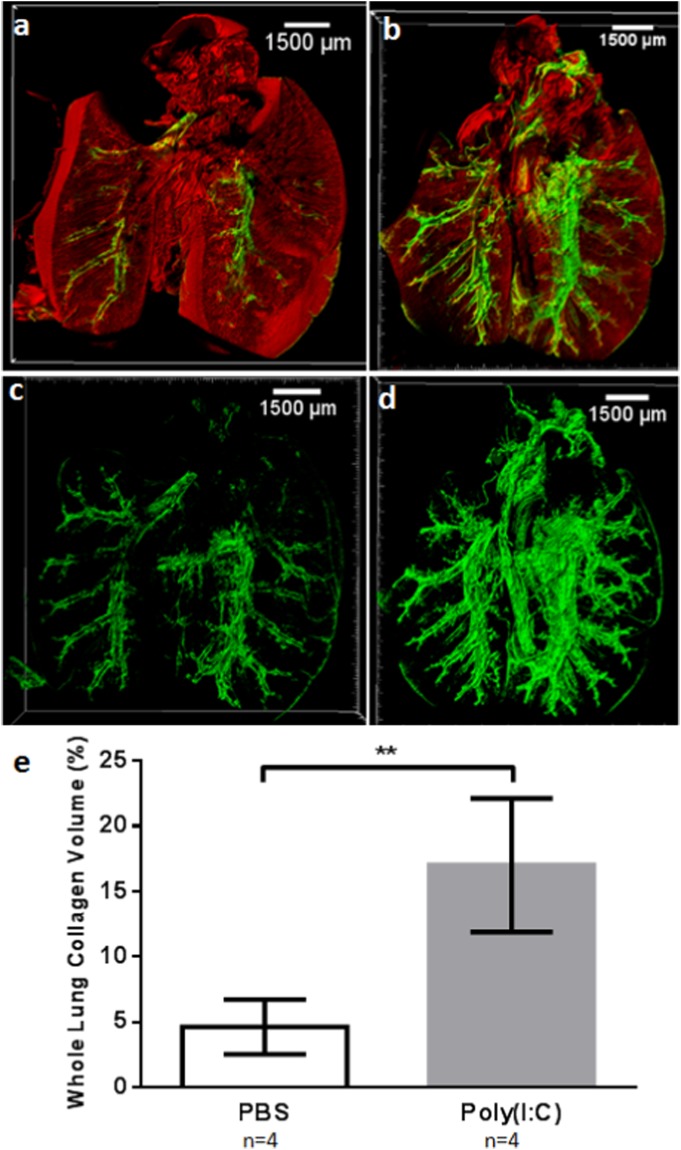
Quantification of Whole Lung Fibrosis Induced by poly(I:C) Treatment and Imaged by MPM/SHGM Mice administered intranasal doses of poly(I:C) or PBS every other day for a total of 15 administrations were imaged following the final dose. (**a**) High resolution MPM (red) and SHGM (green) in the PBS treatment group consists primarily of MPM autofluorescence which delineates the structures of airways and vessels with a component arising from SHG from fibrillar collagen surrounding airways and potentially vessels. (**b**) In poly(I:C) lungs SHGM is significantly increased throughout in both lobes compared to PBS treated with similar MPM. Images (**a**,**b**) are 3D coronal cross-sections with proximal planes removed to highlight the SHG component of the airways. 3D SHG reconstructions depicting fibrillar collagen in PBS (**c**) and poly(I:C) (**d**) treated lungs. Quantification of whole collagen distribution revealed significantly higher collagen deposition in poly(I:C) treated lungs compared to PBS treated (**e**).

Volumetric (3D) reconstructions (coronal cross-sections are shown so SHG within the organ can be visualized easily) of PBS- (Fig. 5a,c) and poly(I:C)-treated (Fig. 5b,d) lungs reveal full organ structural differences between the groups, particularly differences in collagen deposition. Co-registration of MPM with SHGM defines the distribution of SHG throughout the lung structures (Fig. 5a,b), while single green channel (SHGM) volumes (Fig. 5c,d) reveal the extent of fibrosis compared to PBS controls. To quantitatively describe the degree of fibrosis in the poly(I:C) model, whole lung collagen content was quantified by calculating the volume occupied by full lung SHG and normalized to whole lung as described above. SHG signal was substantially greater throughout poly(I:C) treated lungs than in the PBS group (Fig. 5e), providing a quantitative indication of fibrosis.

### Airway Specific Collagen deposition

**Figure 6:**
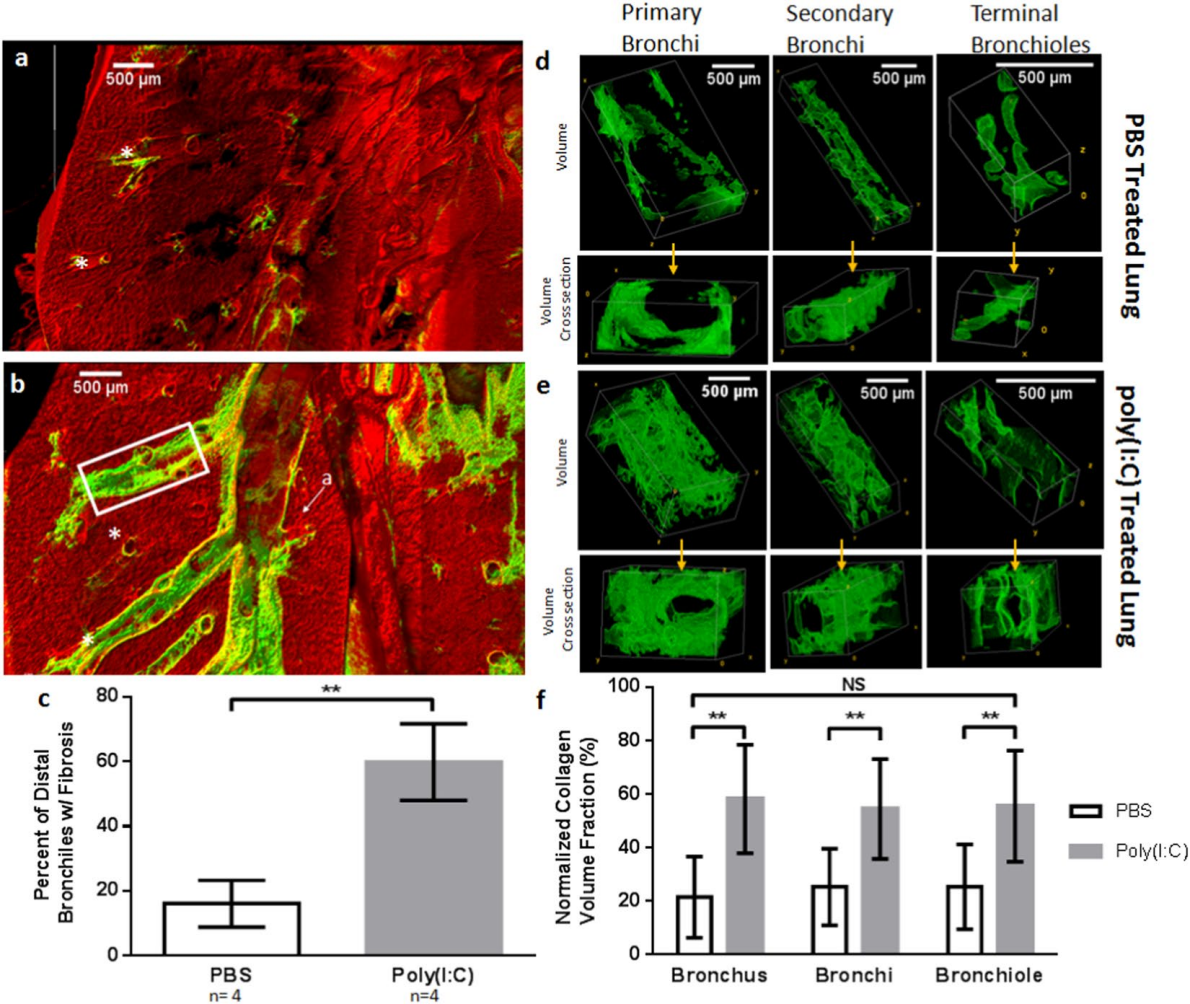
Airway Specific Quantitative Analysis of Subepithelial fibrosis. Coronal cross-sections of lobe segments in PBS treated lungs (**a**) and poly(I:C) treated lungs (**b**). (C) Poly(I:C) lungs showed a higher percentage of distal bronchioles (denoted by *) with visible collagen deposition than PBS controls. (**d**–**e**) 3D reconstructions of the collagen surrounding airway segments. Airway segments comprised non-branching sections of primary bronchi, secondary bronchi, and terminal bronchioles. Alveoli are seen as small circular structures in the MPM channel as denoted by “a” and an example of secondary bronchi segment is shown by the box in (**b**) which were isolated as described in the methods section. (**f**) Volume analysis of airway segments show significantly higher volume fraction of collagen surrounding conduits of poly(I:C) lungs, but no difference between airway type per treatment group. ANOVA: **p < 0.01.

While it is of value to explore the volume of collagen throughout the full lung as a measure of fibrosis, a measure not provided by traditional tools to study fibrosis, it is also of interest to examine differences within finer structures. Thus, the amount of collagen was evaluated for specific airway types throughout the lungs (primary bronchi, secondary bronchi, terminal bronchioles) as shown in Fig. 6. Figure 6a,b show close-up views from full lung reconstructions (coronal cross-sections are shown) demonstrating higher collagen is associated with airways in the fibrosis model (Fig. 6b) than the control (Fig. 6a) acquired and displayed with the same gain/contrast parameters. In a qualitative assessment, a visual count (Fig. 6c) was made of the number of distal bronchioles seen in rotations of a 3D volume that were surrounded by collagen as a percentage of total number of distal bronchioles in each lung. Approximately 60% of distal bronchioles were associated with collagen deposition in the poly(I:C) group, while 16% were associated with collagen in the PBS group (Fig. 6c, n = 4/group; p < 0.001, two-tailed t-test). A separate quantitative analysis of SHG volume fraction per sampled segment was performed of isolated airway segments (primary bronchus, secondary bronchi, and terminal bronchioles) comparing airways of same type/ diameters with examples shown (Fig. 6d–f). The panels shown in Fig. 6d,e are representative SHG 3D volumes segment regions of interest (for each group, the top row shows the segment longitudinal view and the bottom row a transverse view showing the airway opening). These SHG volumes represent the airway subepithelial collagen, so openings in transverse views are not the full airway lumen. Poly(I:C) treated airways of all three types showed higher collagen deposition compared to that of PBS, all with similar degree increase in volume fraction (Fig. 6f). Finally, for each treatment group, we evaluated whether there were differences in fibrosis around airways between the left and right lungs. Results showed no significant difference between airways of a given type when comparing airways from right and left lobes within each treatment group (Supplementary Table I).

**Figure 7:**
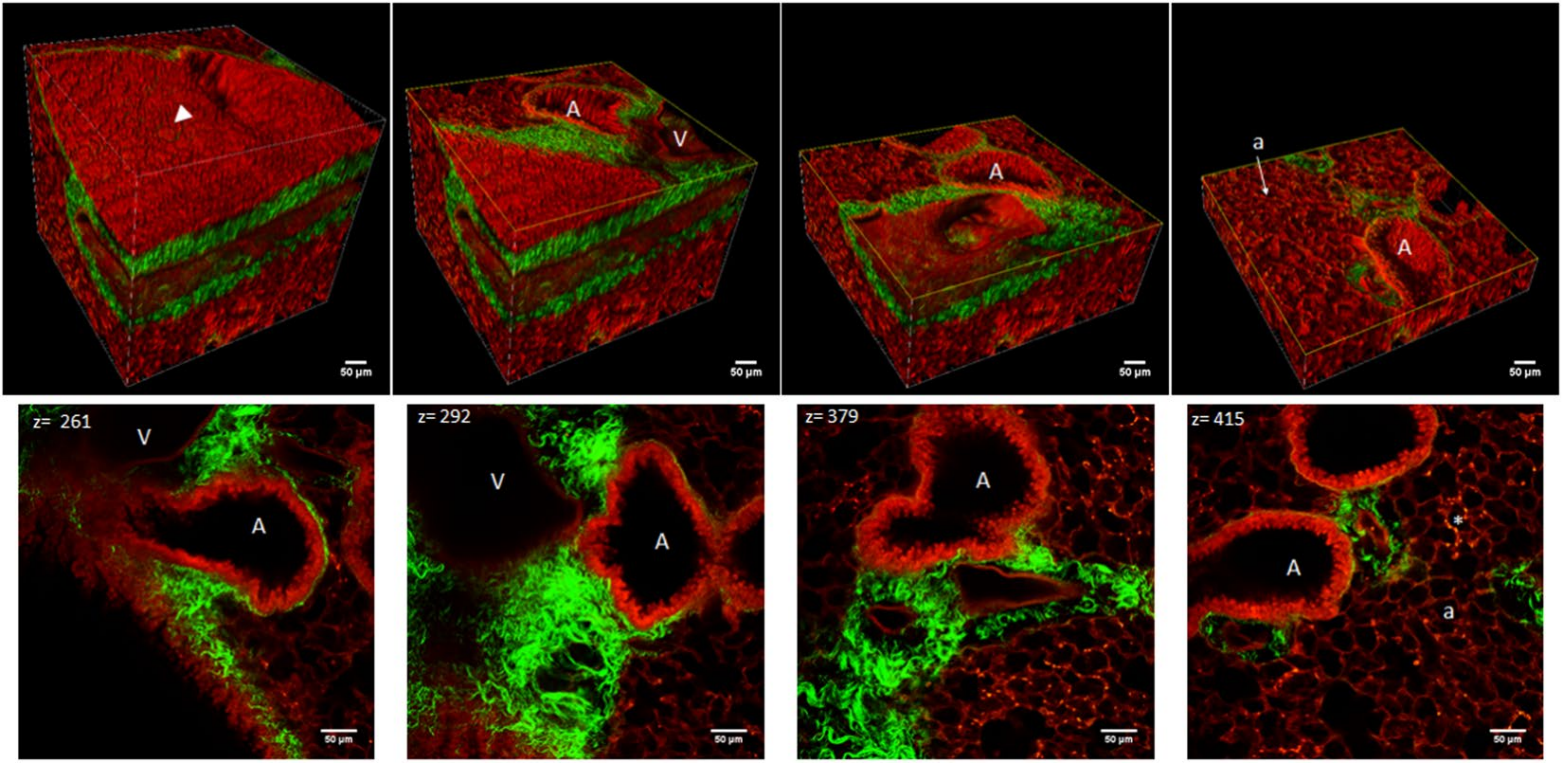
A representative 25x volume of poly(I:C) treated lung. Top row: 3D reconstruction of a volume at the primary bronchus surface extending 450um in depth with a step size of 2 um and shown with cuts at various depths to reveal airway and vascular structures. Bottom row: single plane 25X (1.0 NA) micrographs at various depths. Structures are denoted as Airway(A), Vessel (V), Alveoli (a), Infiltrates (*) are seen as small bright puncta; Arrowhead denotes bronchi epithelium with individual epithelial cells visible on the surface.

An example of a 25x acquisition in the poly(I:C) fibrosis model is shown in Fig. 7. The top panel represents the 3D reconstruction (Imaris) of a region of the lung acquired within the lung volume starting at the surface of a primary bronchus and proceeding in depth to 450 µm. The same volume is shown with cuts made at various depths to reveal underlying structures of airway, vasculature, and collagen. Inflammatory infiltrates shown by puncta surrounding alveoli in the red MPM channel can also be appreciated, though cannot be specifically subtyped due to the nonspecific autofluorescence signal.

## Discussion

There remains a lack of understanding of the pathogenesis and molecular mechanisms that drive pulmonary fibrosis. A gap in the field has been lack of methods for direct mapping of collagen deposition throughout the murine lung. This study demonstrates the ability to visualize and evaluate fibrillar collagen throughout the entirety of the murine lung with microscopic resolution applied for study of whole lung fibrosis. No known previous imaging studies have provided the ability to specifically evaluate the deposition of fibrillar collagen within the entirety of the murine lung or quantitatively evaluated fibrosis. Traditional imaging methods that have been applied for the study of fibrosis either do not provide specific contrast for fibrillar collagen comprising fibrotic areas (e.g. xray, microCT, microPET, IVIS) or require sectioning or destruction of tissue to evaluate collagen content (histology, molecular biology techniques such as western blot analysis). To achieve full adult murine lung collagen and fibrosis imaging our study combined and optimized approaches in lung optical clearing with large scale, label-free MPM/SHGM. OC has previously been shown to result in full lung transparency, allowing imaging of entire lobes labeled with a fluorescent contrast agent^23,30^ or segments of lung based on endogenous signals^21,22^, with no known OC study imaging and quantifying collagen deposition across the entire lung, shown here in an adult mouse model of lung fibrosis.

The approach for lung OC was optimized to include several important steps to maintain lung shape and to avoid major size deviations of tissue reported in previous OC studies, including those of the lung while achieving full transparency and full lung microscopy without compression of the tissue. OC was based on methanol dehydration with clearing by BABB, chosen due to simplicity of the protocol and rapid clearing which occurred within minutes after treatment with BABB. To maintain the shape and avoid collapsing of the lungs, perfusion with PBS and the fixative solution with lung inflation was employed (Fig. 1). An important area of optimization was a modified dehydration procedure that resulted in minimal change in lung size in contrast to other OC studies of lung, in which lungs shrink up to 20–25% with solvent based methods including methanol/BABB^23,24,30^ or expand with CLARITY methods^27,28^. We did this with a gradual graded concentration infusion of lungs with methanol which resulted in gradual dehydration of the tissue, and found minimal tissue size change (+1% increase after graded methanol and 6% after BABB along the horizontal dimension). The methanol dehydrating agent and BABB OC solution were infused into the airway through the cannulated trachea to ensure direct exposure of the internal airway surfaces to dehydration and clearing solutions (Fig. 1). Finally, care was taken to remove all air microbubbles which could contribute to light scattering in the lung – this was done by placing the sample in a vacuum chamber^21^. Physical compression of the sample accomplished bubble removal in another study, but was not used here as we avoided compressing the tissue. We note that in our early trials, extracted adult lungs that were simply immersed in methanol/BABB without infusion^23^ did not clear throughout the entire lung, possibly because lungs in this study were from 18 week mice versus 6–10 week mice. Also in early pilot trials, we began the dehydration step with 50% methanol, followed by 100% methanol as in previous studies using BABB for clearing^23,30^ but we observed significant shrinkage of lungs. These observations in early pilot trials prompted the improvements described.

As indicated, the choice of methanol dehydration with BABB clearing was influenced by the simplicity and low cost of solvent based clearing and rapid clearing which largely occurred within minutes after treatment with BABB. The full lung processing took one day following fixation an advantage over other OC methods of CLARITY which may take up to two weeks^28^ and reported by one study to result in suboptimal clearing^24^, or resin based approaches^21^ which are more complex. Other immersion methods applied in the clearing of lungs, such as DBE^24,30^, do match similar refractive indices to BABB, however, a reagent (Tetrahydrofuran, THF) used in the dehydration step of DBE optical clearing may form explosive crystals. We therefore choose BABB because of potential safety concerns.

Complete transparency of lungs (Fig. 2b,c), combined with the use of several millimeter long working distance objectives, enabled imaging without the need for further tissue processing such as cutting/sectioning, deflation, or flattening. This is in contrast to previous studies in which the infusion steps outlined above were not used (though clearing by BABB was a similarity) and in which confocal microscopy required separation of lobes and trachea with further sample flattening to accommodate the 2 mm working distance^23^. It is noted that while previous efforts in full lung microscopy facilitated by OC used young mice aged 5–10 weeks old^23,29^ or 12 weeks old in optical tomographic imaging^21^, in the current study adult mice aged 22 weeks at the time of optical clearing were used, demonstrating that full lung optical clearing for studies of fibrosis and other disease characteristics are applicable to adult mice with larger organs.

Scott *et al*. first demonstrated whole lobe microscopic imaging and used BABB clearing, with confocal imaging using fluorescent casting of airways and fluorescent antibody labeling of nerve structures^23^. In the label-free imaging used in this study, signal contrast was provided solely from the endogenous optical characteristics of autofluorescence (MPM) and collagen second harmonic generation (SHG) (e.g. Figs 2d, 3). These endogenous signals revealed the complex airway structure as well as the subepithelial collagen matrix throughout the entire volume of the lung. An enhancement in collected signals was likely aided by a mirror placed below the sample, particularly highly forward propagating SHG, a technique adapted from the literature^32^. Results using a 4x objective demonstrate the ability to delineate fine structures of the airway from alveoli seen in MPM micrographs to subepithelial collagen along with small airways and, through the use of mosaicking and image stack stitching, provide a global view of these key structures. Individual alveoli and subepithelial collagen deposition were revealed at 4X (e.g Figs 2d, 6) which provided 2.2 µm resolution and in greater detail at 25x providing 0.4 µm resolution which also provided close detail of airway epithelial lining and vascular endothelial lining (Figs 4a,b and 7). Label-free MPM/SHGM have been applied to native lung and a variety of other tissues to reveal structures several hundred microns in depth^17,19,35,36^ and include studies in lung fibrosis of uncleared lungs^18^. A variety of approaches to evaluate collagen revealed by SHGM exist and include algorithms for evaluating organization and content^17–20^. We did not note collagen orientation differences between treatment groups and with an interest in deposition, we chose volumetric analysis to provide an indication of collagen deposition. It may be of interest to explore more complex organization analyses in future studies. It may also be of interest to explore the combination of SHGM with fluorophores associated with key structures or macromolecules imaged with multiphoton fluorescence. Possibilities for whole lung labeling include vessels, airways, and nerves as introduced by Scott *et al*., or targeted fluorophores for immune cells as explored by Cuccarese *et al*. who used CUBIC tissue clearing in lung carcinoma^29^.

For this study, we used a fibrosis model driven by chronic TLR3 activation induced by repetitive administration of the poly(I:C) agonist, the model representative of a viral-induced fibrosis. This innate epithelial activation model has been shown to cause significant, chronic airway fibrosis and remodeling within a 30 day period^31^. Previous assessment of this model involved histology of regional areas of the lung. As discussed above histology provides a highly limited sampling of tissue areas relative to the full organ. Thus, while previous investigation indicated fibrosis in this model, the nature of this fibrosis including volumetric distribution throughout the lung was unknown. The methods employed here allowed evaluation of collagen deposition throughout the lungs in their entirety, with ultra-deep imaging up to the full thickness of intact murine lungs (up to 4 mm in depth). Results indicated widespread subepithelial fibrosis throughout the volume of the lung of the poly(I:C)-treated mice compared to the PBS group, as observed in Figs 3 and 5 with the differences quantified in (Fig. 5). Masson’s trichrome staining confirmed collagen deposition (Fig. 4), indicated by MPM/SHG imaging.

Beyond full volumetric analysis we evaluated subepithelial collagen deposition in airways according to type, with both macroscopic and microscopic assessment made possible in the same acquisitions (Fig. 6). In a qualitative assessment in which we graded the most distal bronchioles for visible collagen deposition, 60% of the distal bronchioles showed a positive SHG (collagen) signal in the fibrosis model vs. only 16% with visible SHG in the PBS treated group. This provided an indication that fibrosis reached the most distal bronchioles in this model. We then quantified subepithelial collagen volume in the main bronchi in lobes, secondary bronchi, and bronchioles separately. We found that these three airway types experienced fibrosis in the poly(I:C) group, and increases in collagen deposition were of comparable percentage increase (Fig. 6f). Finally, quantitation indicated no significant difference in collagen deposition between lobes within each group (any appearance of increased collagen deposition in right lungs in 3D reconstructions or z-projections are due to the right lung being multilobed (Fig. 2d). Overall, these findings indicate that distribution of pathology in this model extends to the distal airways, a sign of distribution of the intranasal-delivered poly(I:C) beyond proximal airways. A major controversy in asthma and COPD has been the relative contributions of proximal versus distal airway involvement and their relationship to functional impairment of the lung. Because the methods here enables quantitation of fibrosis throughout the entire murine lungs, they can be used study patterns of fibrosis. This includes study of distribution patterns in the variety of models of airway remodeling such as the bleomycin-induced lung injury model mediated by direct epithelial injury, or allergen-induced remodeling mediated by immune cell-secreted fibrogenic cytokines.

In conclusion, this study presents a new method by which fibrosis may be studied across the whole murine lung, addressing a significant gap in the field for visualizing fibrosis in this organ. This capability will be a powerful tool for preclinical studies of fibrosis that can be coupled with other traditional approaches such as whole body imaging by xray, CT, PET or IVIS which do not provide direct measure of collagen but which can provide *in vivo* functional measures prior to lung extraction for methods described here. OC in combination with whole lung MPM/SHGM provided global assessment of lung fibrosis, avoiding regional bias. To our knowledge, this is the first study to quantify fibrillar collagen in the bronchial subepithelial space throughout the entirety of the lung with microscopy. Micro- and macro- structural anatomical components can be studied with this application, making it a useful method to investigate the effects of pulmonary diseases.

### Ethical Approval

Animal experiments were performed according to the NIH Guide for Care and Use of Experimental Animals and approved by the University of Texas Medical Branch (UTMB) Institutional Animal Care and Use Committee (approval no. 1312058A).

## Electronic supplementary material

Supplementary Material

## Data Availability

Data will be made available upon reasonable request.
