# Supplementary material for: Imaging of Murine Whole Lung Fibrosis by Large Scale 3D Microscopy aided by Tissue Optical Clearing
Source: Sci Rep. 2018 Sep 6;8:13348. doi: 10.1038/s41598-018-31182-2 (PMC6127188; doi:10.1038/s41598-018-31182-2)
Supplement: Supplementary file 1 — Supplementary Material [file 41598_2018_31182_MOESM1_ESM.docx]

**Imaging of Murine Whole Lung Fibrosis by Large Scale 3D Microscopy aided by Tissue Optical Clearing**

Authors: Lorenzo F. Ochoa^1,7^, Alexander Kholodnykh^1^, Paula Villarreal^1^, Bing Tian^2,3^, Rahul Pal^1^, Alexander Freiberg^4^, Allan R. Brasier^5^, Massoud Motamedi^1,6^, *Gracie Vargas^1,7^

**Supplementary Material**

**Supplementary Table I. Comparison of collagen deposition in left and right lung airways within each treatment group.**  Analysis of collagen volume associated with each airway type, measured by SHG volume, was compared between the left and right lungs. Airways categorized as primary bronchi, secondary bronchi, and bronchioles were compared between the left lung and right lung for each treatment group. No statistical difference was shown between airways of the right lung and left lung for each treatment group for each airway type. Statistical analysis between right and left lung airways was by two-tailed t-test.

|  | **Airway type** | **Left Lung**  **Ave (stdev)** | **Right Lung**  **Ave (stdev)** | **p-value** |
| --- | --- | --- | --- | --- |
| **PBS Group** | **Primary Bronchi** | 20.3 (13.3) | 22.4(16.3) | 0.74 |
|  | **Secondary bronchi** | 26.0(13.7) | 24.3(14.3) | 0.78 |
|  | **Bronchioles** | 24.4 (14.8) | 26.0 (16.2) | 0.81 |
| **Poly(I:C) Group** | **Primary Bronchi** | 53.7 (21.3) | 62.5(19.2) | 0.29 |
|  | **Secondary bronchi** | 52.5(14.7) | 56.2(21.5) | 0.64 |
|  | **Bronchioles** | 54.4(20.2) | 53.4(20.4) | 0.82 |
